# Epidemiology of acromegaly: review of population studies

**DOI:** 10.1007/s11102-016-0754-x

**Published:** 2016-10-14

**Authors:** Aikaterini Lavrentaki, Alessandro Paluzzi, John A. H. Wass, Niki Karavitaki

**Affiliations:** 1 Endocrine Unit, ARETAIEION Hospital, Faculty of Medicine, National and Kapodistrian University of Athens, Athens, Greece; 2 Department of Neurosurgery, University Hospitals Birmingham NHS Foundation Trust, Birmingham, UK; 3 Oxford Centre for Diabetes, Endocrinology and Metabolism, Churchill Hospital, Oxford, UK; 4 Institute of Metabolism and Systems Research (IMSR), College of Medical and Dental Sciences, University of Birmingham, IBR Tower, Level 2, Birmingham, B15 2TT UK; 5 Centre for Endocrinology, Diabetes and Metabolism, Birmingham Health Partners, Birmingham, UK

**Keywords:** Acromegaly, Epidemiology, Prevalence, Incidence

## Abstract

Acromegaly is a rare condition necessitating large population studies for the generation of reliable epidemiological data. In this review, we systematically analysed the epidemiological profile of this condition based on recently published population studies from various geographical areas. The total prevalence ranges between 2.8 and 13.7 cases per 100,000 people and the annual incidence rates range between 0.2 and 1.1 cases/100,000 people. The median age at diagnosis is in the fifth decade of life with a median diagnostic delay of 4.5–5 years. Acral enlargement and coarse facial features are the most commonly described clinical manifestations. At the time of detection, most of the tumors are macroadenomas possibly relating to diagnostic delays and posing challenges in the surgical management. Increased awareness of acromegaly amongst the medical community is of major importance aiming to reduce the adverse sequelae of late diagnosis and treatment, improve patient outcomes and, hopefully, reduce the burden on the health care system.

## Introduction

Acromegaly is a rare condition characterized by growth hormone (GH) excess and elevated Insulin-like growth factor 1 (IGF-I) levels attributed in the vast majority of cases, to a pituitary adenoma. Mortality is high in uncontrolled disease and adequate biochemical control may restore it to normal [1].

The presentation of acromegaly can be insidious and despite the advances in the field, there are significant diagnostic delays with adverse sequelae on the prognosis of the patients. The recently published Endocrine Society clinical practice guidelines suggest screening for acromegaly by measurement of IGF-I in patients with typical clinical manifestations, but also in those who lack the typical clinical picture and have several associated conditions (sleep apnea syndrome, type 2 diabetes mellitus, debilitating arthritis, carpal tunnel syndrome, hyperhidrosis, and hypertension) [2]. The impact of this approach on the prevalence and incidence rates of acromegaly in the future remains to be elucidated.

Accurate and up-to-date epidemiological data on acromegaly are of major importance for describing patterns of disease and generating hypotheses on causal factors, for assessing the impact of this condition and its co-morbidities on patients, families and the community, for evaluating the burden of acromegaly on the health care system and for providing guidance on optimal allocation of resources (clinical and research) which will ultimately lead to improvement of patient outcomes.

In this review, we will systematically analyse the epidemiological profile of acromegaly based on recently published population studies from various geographical areas. Data from cancer registries or isolated tertiary referral centres will not be assessed; the former tend to suffer from under-reporting of benign tumours including pituitary adenomas and the latter are affected by selection bias and wide variations in the referral patterns across the world.

## Epidemiology of acromegaly

The population studies assessing the epidemiology of acromegaly are shown in Table 1.

**Table 1:** Prevalence, incidence and age at diagnosis of acromegaly in population studies

Reference	Population covered	Prevalence (per 100,000)			Annual incidence (per 100,000)			Age at diagnosis (years) Median (range)		
		Total	Males	Females	Total	Males	Females	Total	Males	Females
Fernandez et al. [3]	81,449	8.6	4.9	3.7	NA	NA	NA	47 (30–63)	48.5 (30–52)	45 (39–63)
Daly et al. [4]	71,972	12.5	8.3	4.2	NA	NA	NA	47 (17–65)	41 (19–65)	56 (17–63)
Tjornstrand et al. [5]	1,590,640	3.3	1.7	1.6	0.4	0.4	0..4	NA	NA	NA
Agustsson et al. [6]	321,857	13.7	9.0	4.7	NA	0.8	0.4	45 (4–83)	45.0 (15–83)	44 (4–75)
Hoskuldsdottir et al. [7]	316,075	13.3	NA	NA	0.8	NA	NA	44.5^a^ (24.5–49.7)	NA	NA
Raappana et al. [8]	722,000 –733,000	NA	NA	NA	0.3	0.4	0.3	40.5 (12–69)	41	38
Dal et al. [9]	5,534,738	8.5	NA	NA	0.4	NA	NA	48.7^a^ (47.2–50.1)	NA	NA
Bex et al. [10]	10,850,000	4	NA	NA	0.2	NA	NA	NA	42 (8–81)	46 (17–80)
Mestron et al. [11]	Population of Spain in 2001	3.4	NA	NA	0.2	NA	NA	45^a^	NA	NA
Gruppetta et al. [12]	417,608	12.4	10.6	14.3	0.3	0.2	0.4	44 (19–69)	36.5 (19–69)	49.5 (28–68)
Burton et al. [13]	50,170,946	7.8	7.7	7.7	1.1	1.0	1.2	41^a^	NA	NA
Kwon et al. [14]	48,456.369	2.8	1.3	1.5	0.4	NA	NA	44.1^a^	42.2^a^	45.5^a^

*NA* not available

^a^Mean

### Details of populations studied

Most of the published population studies have been conducted in Europe. Fernandez et al. [3] performed a community based cross-sectional study through a computer database search in fourteen General Practice surgeries covering the urban and rural areas of Banbury (Oxford, UK). Daly et al. [4] completed a cross-sectional, case-finding study covering three regions in the Province of Liege, Belgium. The patients were identified by general practitioners and relevant specialists working in public/private practice and further information was sought from hospital case files or other relevant clinical records. Tjornstrand et al. [5] identified patients from the Swedish Pituitary Registry and medical records from six hospitals in a county of western Sweden. Agustsson et al. [6] performed a nationwide population study in Iceland and information was obtained from medical records at the National University Hospital, three district hospitals, all privately practicing endocrinologists and gynaecologists in the country, all radiology departments in Iceland, the Icelandic Cancer Registry, the department of pathology and the Icelandic Heart Association. Hoskuldsdottir et al. [7] collected data from the medical records at the National University Hospital in Iceland, the largest hospital out of the capital, the largest private outpatient clinic in Iceland and from all endocrinologists treating adult patients in the country. Raappana et al. [8] obtained information from Oulu University Hospital in which the great majority of patients with pituitary tumor from the four northernmost provinces of Finland are referred. Dal et al. [9] covered the entire population of Denmark and obtained data from the Danish National Patient Registry, the Danish Civil Registration System and the Danish Register of Causes of Death. Bex et al. [10] collected information on patients with acromegaly through a nationwide survey involving all endocrinologists managing patients with pituitary disorders in Belgium and the Grand Duchy of Luxembourg (the University Hospital of Liege and some local centers did not take part). Mestron et al. [11] analyzed data from the Spanish acromegaly registry in which patients were voluntarily registered by the managing physicians. Gruppetta et al. [12] carried out a thorough search for patients with pituitary adenoma in the central hospital registries covering the Maltese islands. In the Burton et al. [13] study, the source population was derived from a large health insurance database which contains medical and pharmacy claims, and enrollment information from a geographically diverse group of health plans in the USA. The main limitation of this study is that only cases with a related medical claim were captured leading to possible underestimation of true prevalence. Finally, Kwon et al. [14], in a nationwide survey in South Korea selected 74 secondary and tertiary care hospitals where patients with acromegaly were diagnosed or treated by both endocrinologists and neurosurgeons.

In the above studies, the sources of information used tend to be extensive and may be sufficient for the identification of the majority of the relevant cases. However, the impact of each national health system, the referral pathways and policies, the accuracy of reporting/registering patients in national databases and the extend of involvement of the private sector in the diagnosis and management of subjects with acromegaly need to be taken into account.

### Prevalence, incidence, sex distribution and age at diagnosis

The total prevalence ranges between 2.8 and 13.7 cases per 100,000 people [3–14]. The highest rates have been reported in two studies covering Iceland [6, 7] and in one from the Maltese islands [12], whereas the lowest has been found by Kwon et al. [14], Tjornstrand et al. [5] and Mestron et al. [11]. Notably, the last two studies obtained information from the Swedish Pituitary Registry and the Spanish acromegaly registry, respectively, and the possibility of under-reporting cannot be excluded. It should be also pointed out that the two studies from Iceland [6, 7] cover the same population and have a significant overlap in the sources of their data; this could sway the overall results and they should not be considered as independent from each other. In most studies, there is a rather equal distribution of prevalence between males and females, with the exception of Daly et al. [4] and Agustsson et al. [6], in which men showed prevalence rates twice as high as those of women.

The incidence rates range between 0.2 and 1.1 cases/100,000 people and the very small numbers make it difficult to reach conclusions for potential differences between the sexes (males: 0.2–1.0/100,000/year and females 0.3–1.2/100,000/year) [5–14].

Previously published studies (between 1980 and 2001) have reported prevalence between 3.8 and 6.9/100,000 and incidence rates between 0.28 and 0.4/100,000 [15–19]. The higher rates of the contemporary studies may be attributed to the methodology used (source of data: population vs tertiary referral centres, intensive case searching approaches), to the increased awareness of pituitary disease and the advances in the diagnostic tools, to the fact that affected patients may seek medical attention earlier in the last years or they may reflect a true increase in the prevalence.

The median age at diagnosis is similar in all reports—fifth decade of life—and ranges between 40.5 and 47 years (males: 36.5–48.5 and females 38–56) [3, 4, 6, 8, 10, 12].

It is of note that relevant epidemiological data on gigantism or young-onset acromegaly are sparse and this is mainly attributed to the rarity of this entity; Daly et al. [4] and Kwon et al. [14] reported that amongst the patients with acromegaly identified, 22.2 and 2.4 %, respectively were aged between 0 and 19 years. In such cases, and particularly given the recent advances in the genetics of acromegaly, genetic causes need to be considered [20].

### Presentation and diagnostic delay

The frequency of the presenting manifestations has been systematically assessed in two population studies (Table 2). Acral enlargement and coarse facial features are the most commonly described (78.8–85.7 % and 71.2–71.4 %, respectively) followed by headaches, macroglossia, increased sweating, arthralgias, increased skin thickness, snoring, tiredness and carpal tunnel syndrome [3, 7]. Hoskuldsdottir et al. [7] also looked at the presence of comorbidities at diagnosis and reported hypertension in 48 %, diabetes mellitus in 13 %, impaired glucose tolerance in 19 %, heart failure in 10 % and coronary artery disease in 8 % of the cases. It should be noted however, that the reliability of the published information may be affected by the accuracy of documentation during the history taking and this needs to be taken into account when reviewing the frequency of the presenting signs and symptoms.

**Table 2:** Clinical manifestations of acromegaly at diagnosis as reported in population studies

Reference	Presenting manifestations (%)										
	Acral enlargement	Headaches	Coarse facial features/change of facial bones	Macro-glossia	Increased sweating	Skin thickening	Snoring	Arthralgias	Carpal tunnel syndrome	Tiredness	Visual field defects/visual disturbances
Fernandez et al. [3]	85.7	NA	71.4	NA	51.7	NA	28.6	28.6	14.3	14.3	NA
Hoskuldsdottir et al. [7]	78.8	55.8	71.2	46.2	44.2	38.5	NA	50	NA	38.5	34.6

*NA* not available

The duration of symptoms until diagnosis, which may in a large part be due to diagnostic delay, is still considerable, with median estimated intervals of 4.5–5 years; however, delays of 15 or even 25 years have been reported, in accord with the insidious onset of symptomatology (Table 3) [3, 4, 7, 11]. It should be noted though that the actual duration of the disease is not uniformly collected or determined and the relevant information, as provided by the patient, may be subjective and prone to recall bias. Nonetheless, these data highlight the need for enhancing the awareness of acromegaly amongst clinicians aiming to reduce the adverse sequelae of late detection and management.

**Table 3:** Duration of symptoms until diagnosis in acromegaly as reported in population studies

Reference	Duration of symptoms until diagnosis (years) Median (range)		
	Total population	Males	Females
Fernandez et al. [3]	Median 4.5 (range 1.5–15.0)	Median 3.0 (range 1.5–15.0)	Median 6.0 (range 3.0–15.0)
Daly et al. [4]	Median 5 (range 1–25)	Median 5.5 (range 1–25)	Median 3 (range 1.6–5)
Hoskuldsdottir et al. [7]	“for more than 3 years in most cases”	NA	NA
Mestron et al. [11]	“the patients’ estimates of the year in which symptoms began was around 5 years before diagnosis”	NA	NA

*NA* not available

At the time of detection, most cases are macroadenomas (>2/3 of cases); this may relate to diagnostic delays and poses challenges in the surgical management of these tumors (Table 4) [3–6, 8–12, 14].

**Table 4:** Frequency of macro- and microadenomas in patients with acromegaly as reported in population studies

Reference	Adenoma size			
	Macroadenoma (% of cases)		Microadenoma (% of cases)	
	Males	Females	Males	Females
Fernandez et al. [3]	85.7		14.3	
Daly et al. [4]	88.9^a^		0	
	100	66.7^a^	0	0^a^
Tjornstrand et al. [5]	78	77	22	23
Agustsson et al. [6]	62.5	71.4	28.1	23.8
Raappana et al. [8]	78		22	
Dal et al. [9]	69		31	
Bex et al. [10]	79^a^		16^a^	
Mestron et al. [11]	69^a^		25.8^a^	
Gruppetta et al. [12]	73.1		26.9	
	68.2	76.7	31.8	23.3
Kwon et al. [14]	82.9		17.1	

^a^Percentages do not add up to 100 due to cases with unknown tumor size

Epidemiological data on the prevalence of familial acromegaly are limited. Bex et al. [10] identified four patients with MEN1 and two with Familial Isolated Pituitary Adenoma (FIPA)—somatotropinoma in a total of 418 acromegalics giving rates of 0.95 and 0.48 %, respectively. Mestron et al. [11] found three patients with MEN1 in a total of 1219 subjects included in the Spanish Acromegaly Registry (0.25 %).

## Conclusions

The rarity of acromegaly necessitates large population studies for the generation of reliable epidemiological data. In the last few years, a number of reports based on different geographical areas and variable health systems have provided information on the prevalence and incidence of this condition and suggest 2.8–13.7 cases per 100,000 people and 0.2–1.1 cases/100,000 people/year, respectively. Whether these rates may change with the application of screening of patients with acromegaly-associated conditions remains to be clarified. The diagnostic delay is still considerable and the disease is usually confirmed in the fifth decade of life affecting economically active individuals; this translates into loss of productivity, social and financial consequences and long-term burden on the health care system, necessitating increased awareness of this condition in the medical community.

Further areas that remain to be clarified in the epidemiology of acromegaly include possible geographical variations and the impact of other factors (e.g. environmental, ethnic, sex, type of health care system, availability and access to health care resources), as well as data on early-onset and familial acromegaly and on mixed GH-prolactin secreting adenomas. The latter will require adequate powered population collaborative studies which are eagerly awaited in the future.

## References

[1] Ntali G, Karavitaki N (2015) Recent advances in the management of acromegaly. F1000Res. doi:10.12688/f1000research.7043.1 10.12688/f1000research.7043.1 PMC4754011 26918140

[2] Katznelson L, Laws ER, Melmed S, Molitch ME, Murad MH, Utz A, Wass JA, Endocrine Society (2014). Acromegaly: an endocrine society clinical practice guideline. J Clin Endocrinol Metab.

[3] Fernandez A, Karavitaki N, Wass JAH (2010). Prevalence of pituitary adenomas: a community-based, cross-sectional study in Banbury (Oxfordshire, UK). Clin Endocrinol (Oxf).

[4] Daly AF, Rixhon M, Adam C, Dempegioti A, Tichomirowa MA, Beckers A (2006). High prevalence of pituitary adenomas: a cross- sectional study in the province of Liege, Belgium. J Clin Endocrinol Metab.

[5] Tjörnstrand A, Gunnarsson K, Evert M, Holmberg E, Ragnarsson O, Rosén T, Filipsson Nyström H (2014). The incidence rate of pituitary adenomas in western Sweden for the period 2001–2011. Eur J Endocrinol.

[6] Agustsson TT, Baldvinsdottir T, Jonasson JG, Olafsdottir E, Steinthorsdottir V, Sigurdsson G, Thorsson AV, Carroll PV, Korbonits M, Benediktsson R (2015). The epidemiology of pituitary adenomas in Iceland, 1955–2012: a nationwide population-based study. Eur J Endocrinol.

[7] Hoskuldsdottir GT, Fjalldal SB, Sigurjonsdottir HA (2015). The incidence and prevalence of acromegaly, a nationwide study from 1955 through 2013. Pituitary.

[8] Raappana A, Koivukangas J, Ebeling T, Pirilä T (2010). Incidence of pituitary adenomas in Northern Finland in 1992–2007. J Clin Endocrinol Metab.

[9] Dal J, Feldt-Rasmussen U, Andersen M, Kristensen LØ, Laurberg P, Pedersen L, Dekkers OM, Sørensen HT, Jorgensen JO (2016). Acromegaly incidence, prevalence, complications, and long-term prognosis: a nationwide cohort study. Eur J Endocrinol.

[10] Bex M, Abs R, T’Sjoen G, Mockel J, Velkeniers B, Muermans K, Maiter D (2007). AcroBel–the Belgian registry on acromegaly: a survey of the ‘real-life’ outcome in 418 acromegalic subjects. Eur J Endocrinol.

[11] Mestron A, Webb SM, Astorga R, Benito P, Catala M, Gaztambide S, Gomez JM, Halperin I, Lucas-Morante T, Moreno B, Obiols G, de Pablos P, Paramo C, Pico A, Torres E, Varela C, Vazquez JA, Zamora J, Albareda M, Gilabert M (2004). Epidemiology, clinical characteristics, outcome, morbidity and mortality in acromegaly based on the Spanish Acromegaly Registry (Registro Espanol de Acromegalia, REA). Eur J Endocrinol.

[12] Gruppetta M, Mercieca C, Vassallo J (2013). Prevalence and incidence of pituitary adenomas: a population based study in Malta. Pituitary.

[13] Burton T, Le Nestour E, Neary M, Ludlam WH (2016). Incidence and prevalence of acromegaly in a large US health plan database. Pituitary.

[14] Kwon O, Song YD, Kim SY, Lee EJ, Rare Disease Study Group, Science and Research Committee, Korean Endocrine Society (2013). Nationwide survey of acromegaly in South Korea. Clin Endocrinol (Oxf).

[15] Alexander L, Appleton D, Hall R, Ross WM, Wilkinson R (1980). Epidemiology of acromegaly in the Newcastle region. Clin Endocrinol (Oxf).

[16] Bengtsson B, Eden S, Ernest I, Oden A, Sjogren B (1988). Epidemiology and long-term survival in acromegaly: a study of 166 cases diagnosed between 1955 and 1984. Acta Med Scand.

[17] Ritchie CM, Atkinson AB, Kennedy AL, Lyons AR, Gordon DS, Fannin T, Hadden DR (1990). Acertainment and natural history of treated acromegaly in Northern Ireland. Ulster Med J.

[18] Etxabe J, Gaztambide S, Latorre P, Vazquez JA (1993). Acromegaly: an epidemiological study. J Endocrinol Invest.

[19] Davis JR, Farrell WE, Clayton RN (2001). Pituitary tumours. Reproduction.

[20] Rostomyan L, Daly AF, Beckers A (2015). Pituitary gigantism: causes and clinical characteristics. Ann Endocrinol (Paris).

